# Risk factors for patients with acute hospital-acquired symptomatic pulmonary thromboembolism

**DOI:** 10.1038/s41598-023-34589-8

**Published:** 2023-05-09

**Authors:** Lujuan Ye, Hailiang Xie, Minggui Lai, Guofu Zheng, Yuancai Xie, Xiaochun Liu

**Affiliations:** The Department of General Surgery, Ganzhou People’s Hospital, Ganzho, 341000 Jiangxi People’s Republic of China

**Keywords:** Risk factors, Embolism, Thromboembolism, Thrombosis

## Abstract

This study aimed to identify independent risk factors for acute hospital-acquired symptomatic pulmonary embolism (HA-SPE) by comparing the clinical data of HA-SPE and acute nonhospital-acquired symptomatic pulmonary embolism (NHA-SPE). A total of 292 patients were included in the analysis and divided into two groups: 191 patients had acute NHA-SPE, and 101 patients had acute HA-SPE. The average age of these 292 patients was 63.2 years, and the sample included 145 males. Multivariate analysis showed that malignant tumour (OR, 3.811; 95% CI [1.914–7.586], P = 0.000), recent surgery (OR, 7.310; 95% CI 3.392–15.755], P = 0.000), previous VTE (OR, 5.973; 95% CI 2.194 16.262], P = 0. 000), and the length of stay (LOS) (OR, 1.075; 95% CI [1.040–1.111], P = 0.000) were independent risk factors for acute HA-AEP. The c-statistic for this model was 0.758 (95% CI [0.698–0.800], P < 0.0001). The K-M curve showed that the hazard ratio (HR) of the HA group to the NHA group in all-cause mortality was 3.807 (95% CI [1.987, 7.295], P = 0.0061). Strengthening the prevention and control of patients with these risk factors may reduce the incidence of acute HA-SPE.

## Introduction

Acute pulmonary embolism (PE) is currently the third leading cause of death in human vascular diseases^1,2^, mainly in adults. The European Society of Cardiology (ESC) has proposed an updated risk stratification model for death in patients with acute PE (2019 ESC model)^3^. The disease can occur both inside and outside the hospital, and it still imposes a relevant medical and societal burden^4,5^. The prevention and control of venous thromboembolism (VTE), including deep vein thrombosis (DVT) and PE, is an important regulatory task for hospitalised patients^6–8^. Many studies have reported that the risk factors for PE include advanced age, prolonged bed rest, surgery, malignant tumours and trauma. Identifying risk factors will be conducive to the prevention and control of VTE^9–12^. We have understood that acute hospital-acquired symptomatic pulmonary embolism (HA-SPE) and acute nonhospital-acquired symptomatic pulmonary embolism (NHA-SPE) have a similar clinical course but different outcomes.

Based on follow-up data, we found that the mortality rate of HA-SPE was higher than that of NHA-SPE, yet minimal data examining the outcomes of in-hospital and follow-up are available in patients with acute SPE. Therefore, the objective of this study was to identify independent risk factors for HA-SPE by comparing the clinical data of HA-SPE and NHA-SPE.

## Methods

### Ethics and consent statement

This retrospective cohort study was approved by the Medical Ethics Committee of Ganzhou People’s Hospital, and the experiments were carried out in accordance with the approved guidelines. For this retrospective study, informed consent was waived by the Medical Ethics Committee of Ganzhou People’s Hospital.

### Study population

This retrospective cohort study was conducted at a regional medical centre, a 3200-bed general university-affiliated hospital. Data from consecutive acute symptomatic pulmonary embolism (SPE) patients hospitalised in our hospital from January 2018 to December 2020 were collected by clinician review through electronic medical record retrieval to analyse the risk factors for acute hospital-acquired symptomatic pulmonary embolism (HA-SPE).

Inclusion criteria: Patients with a discharge diagnosis of acute PE in their medical records.

The exclusion criteria were as follows: (1) age < 18 years; (2) malignant PE; (3) asymptomatic acute PE; and (4) no computer tomography pulmonary angiography (CTPA) data.

According to the research needs, we collected baseline demographics and variables previously shown to increase the risk of VTE, including gender, age, body mass index (BMI), hypertension, diabetes, previous VTE, malignant tumour, renal insufficiency, coronary heart disease, cerebrovascular disease, chronic obstructive pulmonary disease (COPD), deep vein thrombosis (DVT), trauma, recent surgery, and length of stay (LOS). The first laboratory results of D-dimer, fibrinogen, red blood cells and platelets were collected at hospitalisation. Right ventricular dysfunction (RVD), simplified pulmonary embolism severity index (sPESI) were collected according to the risk stratification of pulmonary embolism proposed by the European Society of Cardiology (ESC). The discharged patients were followed up by telephone. We focused on whether the patient died in the hospital. If death occurred after discharge, the time of death was recorded. All-cause mortality for follow-up periods of at least 24 months for all patients was recorded.

### Definitions

Acute PE is a general term for a group of diseases or clinical syndromes caused by various emboli obstructing the pulmonary artery system, including pulmonary thromboembolism (PTE), fat embolism syndrome, amniotic fluid embolism, and air embolism. PTE is the most common type of PE^13^. The PE studied in this paper refers to PTE.

Acute SPE refers to the sudden onset of the following symptoms: dyspnoea, chest pain and even haemoptysis. These signs may include decreased oxygen saturation and cyanosis and pulse oxygen saturation < 90% without oxygen intake. RVD occurred in some cases. In patients with hemodynamic instability, blood pressure drops, and the patients can even die from shock^14^. This scenario includes patients less than 2 weeks after the onset of symptoms.

Patients admitted to the hospital for other diseases without symptoms of PE at admission, those who developed symptoms of PE after admission and those confirmed to have the symptoms of PE by CTPA were acute HA-SPE patients. Patients with symptoms of acute PE on admission and confirmed by PCTA were acute NHA-SPE patients.

The sPESI score was used to assess the risk stratification of acute SPE. sPESI score was assessed as previously described (Score ≥1 was defined as a high risk of 30 days mortality, and the score of 0 was defined as a low risk)^15^. RVD was defined as a right-to-left maximum dimension ratio ⩾0.9 when measured in the two-dimension axial transverse images at the valvular plane at CT angiography^16^.

### Statistical analysis

SPSS software package version 26.0 (IBM, Armonk, NY, USA), GraphPad Prism 8 (version 8.0.1.244) and MedCalc were used for data analysis and graph drawing. The continuous data of the two groups are described by the mean ± standard deviation, and the independent samples of the two groups were compared by the *t* test. Nonparametric data are expressed as medians (interquartile ranges) and were compared with the use of the Mann‒Whitney *U* test. Categorical data are expressed as percentages and were compared using the χ^2^ test or Fisher’s exact test. GraphPad Prism 8 was used to draw the survival curve of the two groups. Univariate and multivariate logistic regression analyses were used to analyse the risk factors for the disease. Variables with two-tailed P < 0.05 in univariate analysis were included in the multivariate regression model to determine the independent risk factors for acute HA-SPE. Odds ratios (ORs) and 95% confidence intervals (CIs) were reported. The area (c-statistic) under the receiver operating curve (ROC) was calculated and plotted using MedCalc to evaluate the predictive value of the model. All tests were two-sided with a significance level of 0.05.

## Results

### General data of the patients

**Figure 1:**
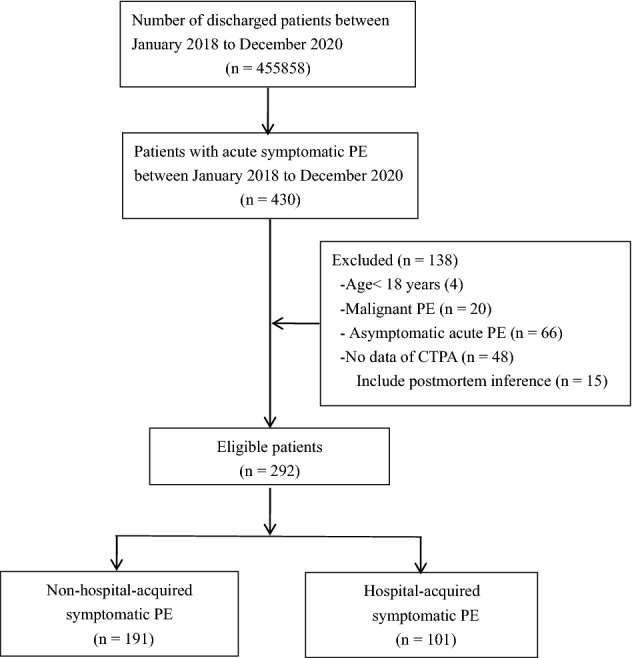
Patient flowchart.

From January 2018 to December 2020, a total of 455,858 patients were discharged from a single centre at our hospital, and 430 consecutive patients with a discharge diagnosis of acute PE were discharged. Among them, 4 patients were younger than 18 years old. Twenty patients had malignant thrombus; 66 patients had asymptomatic acute PE; and 48 patients did not have PCTA, including 15 patients with postmortem inference. The remaining 292 patients were included in the analysis and were divided into two groups according to whether they had acute HA-SPE: 191 patients had acute NHA-SPE, and 101(about 0.02%) patients had acute HA-SPE (Fig. 1). Among 191 patients with acute NHA-SPE, all of them received anticoagulant therapy except 10 patients who had contraindications to anticoagulation. Prophylactic anticoagulation was used in 86 of 101 patients with acute HA-SPE, mechanical prophylaxis was used to the other 15 patients for the bleeding risk.

The average age of the 292 patients was 63.2 years, and the cohort included 145 males. The incidence of DVT, diabetes, hypertension, renal insufficiency, coronary heart disease, cerebrovascular disease or trauma did not significantly differ between the two groups. In total, 71 patients had malignant tumours in the two groups, including 20 in the NHA group and 51 in the HA group, with a significant difference between the two groups (P = 0.000). Moreover, 42 tumour patients died (15 patients died in the hospital, 21.1%); specifically, 8 and 34 tumour patients died in the two groups, respectively (P = 0.040). In addition, 8 cases and 19 patients had a history of VTE in the two groups, respectively, and the difference was also significant (P = 0.000). The number of patients who underwent recent surgery in the two groups was 14 and 46, respectively (P = 0.000). The D-dimer level also significantly differed between the two groups in the first laboratory test results after admission (P = 0.000). In addition, the acute HA-SPE group had a longer hospital stay (P = 0.000). There were significant differences in RVD between the two groups. Patient clinical characteristics are presented in Table 1 (See Supplementary file).

**Table 1:** Baseline characteristics of the patients.

Variable	Total (n = 292)	NHA-SPE (n = 191)	HA-SPE (n = 101)	P value
Gender				0.596*
Male	145 (49.7%)	97 (50.8%)	48 (47.5%)	
Age	63.2 ± 13.5	62.8 ± 13.7	63.8 ± 13.3	0.573^‡^
BMI	23.0 ± 2.9	23.1 ± 2.9	22.9 ± 2.9	0.604^‡^
Comorbidities				
DVT	160 (54.8%)	109 (57.1%)	51 (50.5%)	0.283*
Malignant tumour	71 (24.3%)	20 (10.5%)	51 (50.5%)	0.000*
Diabetes	32 (11.0%)	23 (21.0%)	9 (8.9%)	0.415*
Hypertension	110 (37.7%)	78 (40.8%)	32 (31.7%)	0.125*
Renal insufficiency	42 (14.4%)	26 (13.6%)	16 (15.8%)	0.606*
Coronary heart disease	81 (27.7%)	58 (30.4%)	23 (22.8%)	0.168*
COPD	88 (30.1%)	65 (34.0%)	23 (22.8%)	0.046*
Trauma	41 (14.0%)	27 (14.1%)	14 (13.9%)	0.949*
Cerebrovascular disease	39 (13.4%)	25 (13.1%)	14 (13.9%)	0.854*
Recent surgery	60 (20.5%)	14 (7.3%)	46 (45.5%)	0.000*
Previous VTE	27 (9.2%)	8 (4.2%)	19 (18.8%)	0.000*
d-dimer, median (IQR)	9.8 (2.4, 11.2)	9.8 (4.0, 11.3)	9.8 (1.1, 10.4)	**0.000**^**†**^
Fibrinogen	3.7 (2.6, 4.7)	3.8 (2.9, 4.8)	3.5 (2.3, 4.5)	0.056^†^
Red blood cells(*10^12^/L)	4.3 (3.7, 4.8)	4.4 (3.8, 4.9)	4.1 (3.5, 4.7)	0.051^†^
Platelets(*10^9^/L)	229.4 ± 97.0	233.3 ± 93.2	222.0 ± 103.8	0.342
LOS	14.4 (8.0, 18.0)	12.3 (8.0, 15.0)	18.4 (11.5, 24.5)	**0.000**^**†**^
RVD	154 (52.7%)	88(46.1%)	66 (65.3%)	0.002*

Baseline characteristics of the patients.

*NHA-SPE* nonhospital-acquired symptomatic pulmonary embolism, *HA-SPE* hospital-acquired symptomatic pulmonary embolism, *BMI* body mass index, *DVT* deep vein thrombosis, *COPD* chronic obstructive pulmonary disease, *VTE* Venous thromboembolism, *IQR* interquartile range, *LOS* length of stay.

*Pearson Chi-Square, ^‡^Independent-sample* t* test, ^†^Mann–Whitney *U* test.

Significant values are in bold.

The Table 2 PE prognostic stratification according to ESC stratification model showed that there were significant differences sPESI and ESC 2019 risk category (early mortality risk) between the two groups.

**Table 2:** PE prognostic stratification according to ESC stratification model.

Variable	Total (n = 292)	NHA-SPE (n = 191)	HA-SPE (n = 101)	P value
sPESI				0.022*
High risk score ≥ 1	243 (83.2%)	152 (79.6%)	91 (90.1%)	
Low risk score = 0	49 (16.8%)	39 (20.4%)	10 (9.9%)	
ESC 2019 risk category (early mortality risk)				0.000*
Low	46 (15.8%)	37 (19.4%)	9 (8.9%)	
Intermediate-low	142 (48.6%)	110 (57.6%)	32 (31.7%)	
Intermediate-high	68 (23.3%)	32 (16.8%)	36 (35.6%)	
High	36 (12.3%)	12 (6.3%)	24 (23.8%)	

PE prognostic stratification according to ESC stratification model.

*PE* pulmonary embolism, *ESC* European Society of Cardiology, *RVD* right ventricle dysfunction, *sPESI* simplified pulmonary embolism severity index.

*Pearson Chi-Square.

Significant values are in bold.

### Risk factors for HA-SPE were analysed by modelling

**Figure 2:**
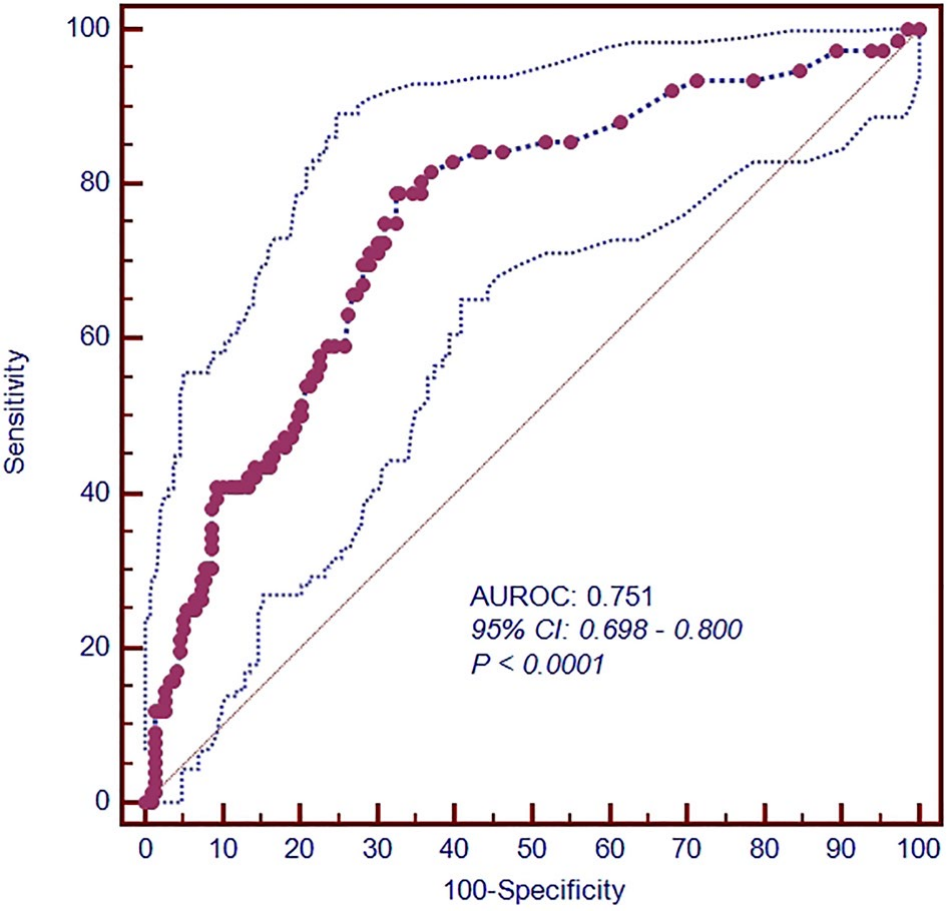
Kaplan–Meier analysis of the all-cause mortality between the two groups.

Age, gender, BMI, malignant tumour, COPD, recent surgery, lower extremity DVT, previous VTE, cerebrovascular accident and LOS were included in the univariate analysis. The P values of malignant tumour, COPD, recent surgery, previous VTE, and LOS were found to be < 0.05. Then, these factors were included in the multivariate analysis by the conditional forwards method, and the results showed that a risk model was established including the four factors. Malignant tumour (OR, 3.811; 95% CI [1.914–7.586], P = 0.000), recent surgery (OR, 7.310; 95% CI 3.392–15.755], P = 0.000), previous VTE (OR, 5.973; 95% CI 2.194 16.262], P = 0.000), and LOS (OR, 1.075; 95% CI [1.040–1.111], P = 0.000) were independent risk factors for acute HA-AEP (Table 3). The c-statistic for this model was 0.758 (95% CI [0.698–0.800], P < 0.0001) (Fig. 2).

**Table 3:** Univariate and multivariate logistic regression analysis of the risk factors related to acute HA-SPE.

Variable	Univariate logistic regression analysis			Multivariate logistic regression analysis		
	OR	95% CI	P value	OR	95% CI	P value
Age	1.005	0.987–1.023	0.572			
Gender	0.878	0.542–1.422	0.596			
BMI	0.978	0.900–1.063	0.603			
Malignant tumor	8.721	4.760–15.979	**0.000**	6.478	3.188–13.163	**0.000**
COPD	0.572	0.329–0.994	**0.047**			**–**
Surgical procedure	10.574	5.409–20.673	**0.000**	6.844	3.110–15.064	**0.000**
DVT	0.767	0.473–1.245	0.284			
Previous VTE	5.300	2.229–12.603	**0.000**	7.165	2.566–20.007	**0.000**
Cerebrovascular accident	1.069	0.529–2.160	0.854			
LOS	1.084	1.051–1.117	**0.000**	1.075	1.040–1.112	**0.000**

Univariate and multivariate logistic regression analyses were used to analyse the risk factors for acute HA-SPE. Variables with two-tailed P < 0.05 in univariate analysis were included in the multivariate regression model.

*HA-SPE* hospital-acquired symptomatic pulmonary embolism, *OR* odds ratio, *CI* confidence interval, *BMI* body mass index, *COPD* chronic obstructive pulmonary disease, *DVT* deep vein thrombosis, *VTE* venous thromboembolism, *LOS* length of stay.

Significant values are in bold.

### All-cause mortality in the hospital and postdischarge of the two groups

**Figure 3:**
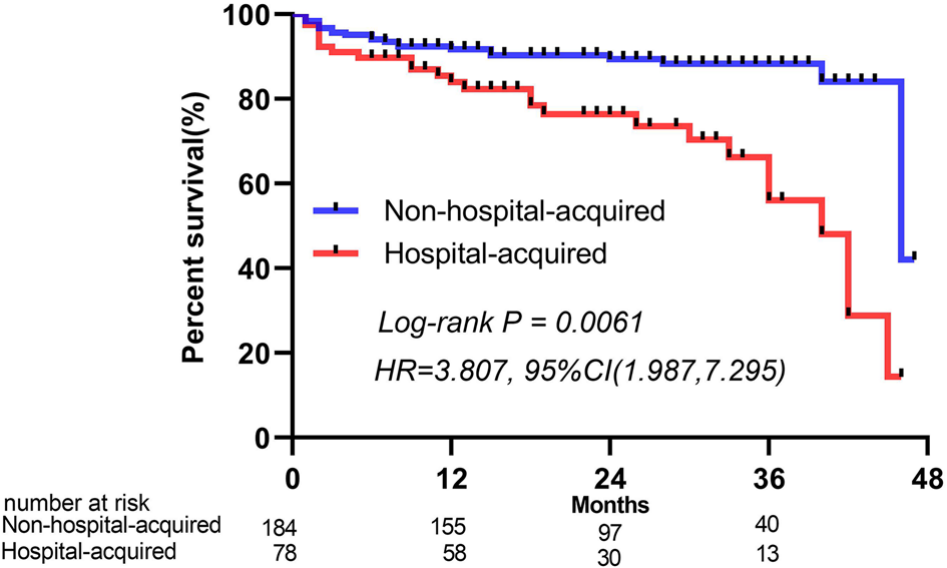
ROC of acute HA-SPE derivation of the model.

The in-hospital all-cause deaths in the NHA group and HA group were 7 and 23, respectively. The in-hospital death rate in the HA group was significantly higher than that in the NHA group (P = 0.000). After a minimum follow-up of 2 years, 21 and 25 out-of-hospital all-cause deaths occurred in the NHA and HA groups, respectively. The total all-cause mortality of the two groups was 14.7% and 47.5%, respectively (P = 0.000). A survival analysis Kaplan–Meier (K-M) curve was used to compare all-cause mortality between the two groups, and the results showed that the hazard ratio (HR) of the HA group to the NHA group in all-cause mortality was 3.807 (95% CI [1.987,7.295], P = 0.0061) (Fig. 3).

## Discussion

In the retrospective analysis comparing the clinical features and follow-up data of acute HA-SPE and acute NHA-SPE, we found that malignant tumour, recent surgery, previous VTE, and LOS were independent risk factors for acute HA-SPE. Studies from this perspective have rarely been reported in the past (Table 3).

Acute PE is a global health problem and can be encountered in all clinical specialties^2,4,5,17^. Approximately 3.1 million new cases are diagnosed in China every year, and this number has been increasing each year^18,19^. Depending on the severity of the embolism, both symptomatic and asymptomatic embolism can occur^20,21^. Acute SPE is a condition we must be on high alert for because it may be life-threatening^9^. Clinicians should always guard against HA-SPE and make it the top priority of VTE prevention and control^22–25^. In this study, we found that acute HA-SPE was associated with a higher risk of in-hospital and out-of-hospital all-cause mortality than acute NHA-SPE. Therefore, the two groups of data need to be compared to determine the risk factors for acute HA-SPE and improve the basis for the prevention and control of VTE. We further established a model and found that malignant tumours, recent surgery, previous VTE, and LOS were independent risk factors for acute HA-SPE.

The demographic results of this study population showed that malignant tumours were more common in patients with acute HA-SPE. Cancer-associated thrombosis is a condition that is increasingly being recognised by physicians and oncologists who manage VTE^21^. In an analysis of 9571 autopsy reports of Dutch cancer patients, Gimbel I. A. et al. found at least one PE event in 1191 autopsies (12.4%; 95% CI 11.8–13.1), including 1074 (90.2%) thromboembolisms, confirming PE as an important complication in cancer patients^26^. Shalaby K. et al. compared noncancer hospitalised patients with cancer hospitalised patients and found that cancer patients hospitalised for PE had higher all-cause in-hospital mortality (11.8% vs. 6.6%, OR 1.79 [95% CI 1.75–1.83]; p < 0.0001), and their results were consistent with ours. Our study included 71 patients with cancer in the two groups, and the in-hospital death rate was 21.1%. In addition, our comparison also found that patients with tumours and acute HA-SPE had a higher mortality rate than those with acute NHA-SPE, which was not mentioned in previous studies.

VTE, including DVT and PE, is a common complication of surgery^27–31^. A total of 60 patients with acute SPE among the consecutive patients underwent surgical treatment, of which 46 (76.7%) were in the HA group. The risk of VTE in surgical patients is determined by both individual predisposing factors and the specific type of surgery^8,32^. Surgery or trauma itself can produce hypobaric hypoxia and activate the coagulation system^33^. The postsurgical inflammatory response, initiated by a cytokine "storm" and occurring within hours of surgery, has been suggested to create a prothrombotic environment that is further exacerbated by several cellular processes, including neutrophil extracellular trap formation, platelet activation, and generation of microparticles bearing tissue factor^34^. Shanafelt Colby et al. studied the clinical characteristics of recent hospitalisation and surgery in acute PE and found that of 2063 patients with acute PE, 633 had a recent hospitalisation and surgery, of whom 319 (50.4%) had a recent surgery^27^.

A previous history of VTE is another risk factor for acute pulmonary embolism in hospitalised patients. In our study, patients with acute HR-SPE had a higher rate of VTE history. Le Gal G. et al. developed a predictive model for acute PE, namely, the Revised Geneva Score. They statistically studied and scored eight clinical indicators for patients presenting to the emergency departments of three European universities with acute PE. In this model, the indicator “Previous VTE” was assigned a score of 3^11^. A study of the risk of VTE in patients with a history of VTE after hospitalisation for surgery suggested that surgery was associated with an increased risk of recurrent DVT/PE in patients with a history of VTE^30,35^. This finding also confirmed that patients with a history of VTE are prone to recurrent VTE in the hospital.

Our current study also found that a long LOS was a risk factor for acute HR-SPE, which can be understood in two ways. First, some patients' primary diseases need a longer hospital stay. Secondly, if acute HR-SPE occurs in the same hospitalisation process, more time for PE treatment were bound to increase the length of hospital stay. Our study also found that the clinic severity of acute HR-SPE was more severe than that of NHR-SPE. Severe PE often requires a longer LOS to complete the treatments^36,37^.

Our findings of risk factors for acute HR-SPE are consistent with the guidelines (ESC 2019). Malignant tumor, surgery, previous VTE and LOS are risk factors for PE. Among which surgery, previous VTE are strong risk factors, malignant tumor is a moderate risk factor, while LOS is a weak risk factor^3^. In addition, our study also found that acute HR-SPE had a higher risk of mortality.

Hospital-acquired VTE is preventable, with interventions including anticoagulants and mechanical measures^38^. However, in our study, 0.02% of patients had acute HR-SPE despite thromboprophylaxis. Our study shows that these patients have had the above independent risk factors and the ESC 2019 risk stratification (early mortality risk) of acute HR-SPE was more severe than that of NHR-SPE. Two studies among hospitalized medically ill patients suggest that a universal approach to prevention has minimal impact on reducing VTE^39,40^. Although optimal strategies for VTE risk assessment and prevention decisions have not been established, clinicians should incorporate VTE and bleeding risk assessment into clinical decision making^41^. This suggests that new clinical trials may be needed to establish further prevention strategies.

Our study has several limitations. First, this study involved only one centre with a relatively small number of patients. Second, this work was a retrospective study using electronic medical record information, and the study population was heterogeneous, which may have introduced a potential risk of information bias. Following up patients’ vital status by telephone may present a risk of subjective bias in the description of patient status. Therefore, a multicentre, prospective, randomised controlled study may be the best way to further understand the risk factors for patients with acute HA-SPE.

## Conclusions

Malignant tumour, surgery, previous VTE and LOS are independent risk factors for acute HA-SPE. Strengthening the prevention and control of patients with these risk factors may reduce the incidence of acute HA-SPE.

## Supplementary Information

Supplementary Information.

## Data Availability

The datasets used and/or analysed during the current study available from the corresponding author on reasonable request.

## Abbreviations

PE: Pulmonary embolism
PTE: Pulmonary thromboembolism
VTE: Venous thromboembolism
SPE: Symptomatic pulmonary embolism
HA-SPE: Hospital-acquired symptomatic pulmonary embolism
NHA-SPE: Nonhospital-acquired symptomatic pulmonary embolism
CTPA: Computer tomography pulmonary angiography
COPD: Chronic obstructive pulmonary disease
BMI: Body mass index
DVT: Deep vein thrombosis
LOS: Length of stay
KM: Kaplan–Meier
ORs: Odds ratios
CIs: Confidence intervals
ESC: European Society of Cardiology
RVD: Right ventricular dysfunction
sPESI: Simplified pulmonary embolism severity index (sPESI)
ROC: Receiver operating curve
HR: Hazard ratio
